# Cell Sheets of Co-cultured Endothelial Progenitor Cells and Mesenchymal Stromal Cells Promote Osseointegration in Irradiated Rat Bone

**DOI:** 10.1038/s41598-017-03366-9

**Published:** 2017-06-08

**Authors:** Huan Liu, Wei Zhou, Nan Ren, Zhihong Feng, Yan Dong, Shizhu Bai, Yang Jiao, Zhongshan Wang, Yimin Zhao

**Affiliations:** 1 State Key Laboratory of Military Stomatology & National Clinical Research Center for Oral Diseases & Shaanxi Key Laboratory of Stomatology, Department of Prosthodontics, School of Stomatology, The Fourth Military Medical University, Xi’an, China; 2 Department of Stomatology, PLA Army General Hospital, 100700, Beijing, China

## Abstract

Irradiated bone has a greater risk of implant failure than nonirradiated bone. The purpose of this study was to investigate the influence of cell sheets composed of co-cultured bone marrow mesenchymal stromal cells (BMSCs) and endothelial progenitor cells (EPCs) on implant osseointegration in irradiated bone. Cell sheets (EPCs, BMSCs or co-cultured EPCs and BMSCs) were wrapped around titanium implants to make cell sheet-implant complexes. The co-cultured group showed the highest osteogenic differentiation potential *in vitro*, as indicated by the extracellular matrix mineralization and the expression of osteogenesis related genes at both mRNA and protein levels. The co-cultured cells promoted ectopic bone formation as indicated by micro-computed tomography (Micro-CT) and histological analysis. In the irradiated tibias of rats, implants of the co-cultured group showed enhanced osseointegration by Micro-CT evaluation and histological observation. Co-cultured EPCs and BMSCs also up-regulated the expression of osteogenesis related genes in bone fragments in close contact with implants. In conclusion, cell sheets of co-cultured EPCs and BMSCs could promote osseous healing around implants and are potentially useful to improve osseointegration process for patients after radiotherapy.

## Introduction

Radiotherapy in combination with surgery is the treatment commonly used for patients with head and neck cancer. Surgical treatment may result in anatomical deformities^1^. Dental implants can be used to replace missing teeth^2^ and anchor facial prostheses^3^, allowing for reconstruction of tumor defects^4^. However, since bone quality is compromised by radiation, the survival of dental implants is often impaired by radiotherapy. The failure risk of implants in irradiated bone is 2–3 times higher than in non-irradiated bone^5^.

Numerous attempts have been made to improve the osseointegration of implants. These measures include surface roughening, chemical modification, surface coating with calcium phosphates and their derivatives or with biomolecules^6^. These methods aim to achieve successful osseointegration by altering cell adhesion, proliferation, gene expression and by controlling cellular responses^7^. However, irradiated bone has compromised regenerative capacity with reduced cellular activity, blood supply and oxygen pressure^8^. In order to improve the osseointegration of implants in irradiated bone, measures need to be taken to improve the biological responses. Hyperbaric oxygen (HBO) treatment may enhance the viability of the irradiated bone by stimulating angiogenesis and lead to improved implant success rates, but its clinical efficacy is still controversial^9^.

Cell sheet engineering has developed as an advanced approach of tissue engineering. It could produce intact sheets of cultured cells along with deposited extracellular matrix (ECM), thus maintaining cell-to-cell and cell-to-ECM connections which are necessary to re-create functional tissues^10^. In our previous research, we used the cell-sheet method to construct a bone marrow mesenchymal stromal cells (BMSC)-implant complex and demonstrated that it had osteogenic ability^11^. Although this method provides a promising technique to improve the osseointegration of implants, it may not be effective in irradiated bone due to the poor blood supply.

Endothelial progenitor cells (EPCs) were initially identified by Isner and Asahara in 1997^12^. EPCs could facilitate new vessel formation by differentiating into endothelial cells, incorporating into neovessels or producing paracrine signals^13^. EPCs have been used for improving bone regeneration in complex bone defects^14^. A recent study showed that EPCs had a critical role in vessel regeneration and functional recovery after whole brain radiation therapy^15^. Therefore, EPCs may participate in vasculogenesis in radiated bone and enhance osseointegration process.

In the present study, cell sheets composed of co-cultured EPCs and BMSCs were used to make cell sheet-implant complexes. We discovered that cell sheets of co-cultured EPCs and BMSCs could promote bone regeneration around implants in irradiated bone using irradiated rat bone model.

## Result

### Characterization of BMSCs

**Figure 1:**
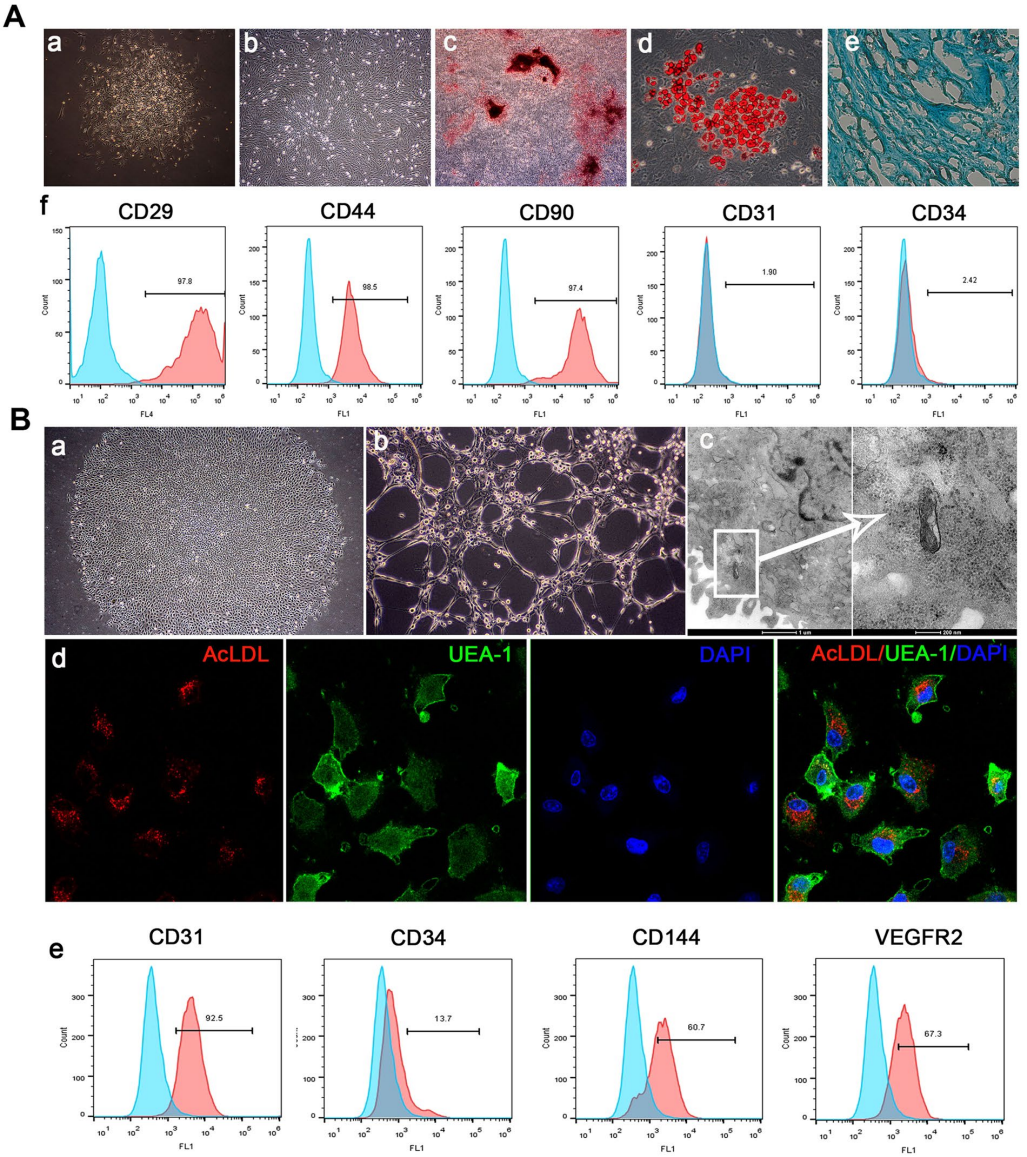
Characterization of BMSCs and EPCs. (**A**) Characterization of BMSCs a: Primary culture of BMSCs (Original magnification x100) b: Subculture of BMSCs (P3, Original magnification x100) c: Mineral node stained with Alizarin Red S (Original magnification x40) d: Fat droplets stained with Oil Red O (Original magnification x200). e: Proteoglycans were stained with Alcian blue (Original magnification x200). f: Flow cytometry analysis of BMSC surface markers. (**B**) Characterization of EPCs a: Primary culture of EPCs (Original magnification x40) b: Lumen formation (Original magnification x100). c: Weibel-Palade body. d: Uptake of DiI-Ac-LDL and FITC-UEA-1. e: Flow cytometry analysis of EPC surface markers.

Primary culture of BMSCs emerged as colonies with spindle-shaped morphology. Cell population appeared to be more homogeneous by the third passage (P3) and showed a swirled pattern. BMSCs expressed high levels of CD29, CD44 and CD90 and were negative for CD31, CD34. BMSCs exhibited multipotent differentiation ability. In osteogenic culture, calcium nodules were stained with Alizarin Red S. In adipogenic culture, intercellular lipid vacuoles were stained with Oil Red O. In chondrogenic culture, proteoglycans were stained with Alcian blue. (Fig. 1A)

### Characterization of EPCs

Primary culture of EPCs emerged as colonies with a cobblestone-like morphology. The cultured EPCs expressed EPC markers CD31, CD144 and VEGFR2 positively. They exhibited tube-like structure when seeded on Matrigel. Weible-Palade bodies, the endothelial specific organelles, were observed in EPCs under the transmission electron microscope. The cells were positive for uptake of both Dil-Ac-LDL and FITC-CEA-1. (Fig. 1B)

### The structure of cell sheets and cell sheet-implant complexes

**Figure 2:**
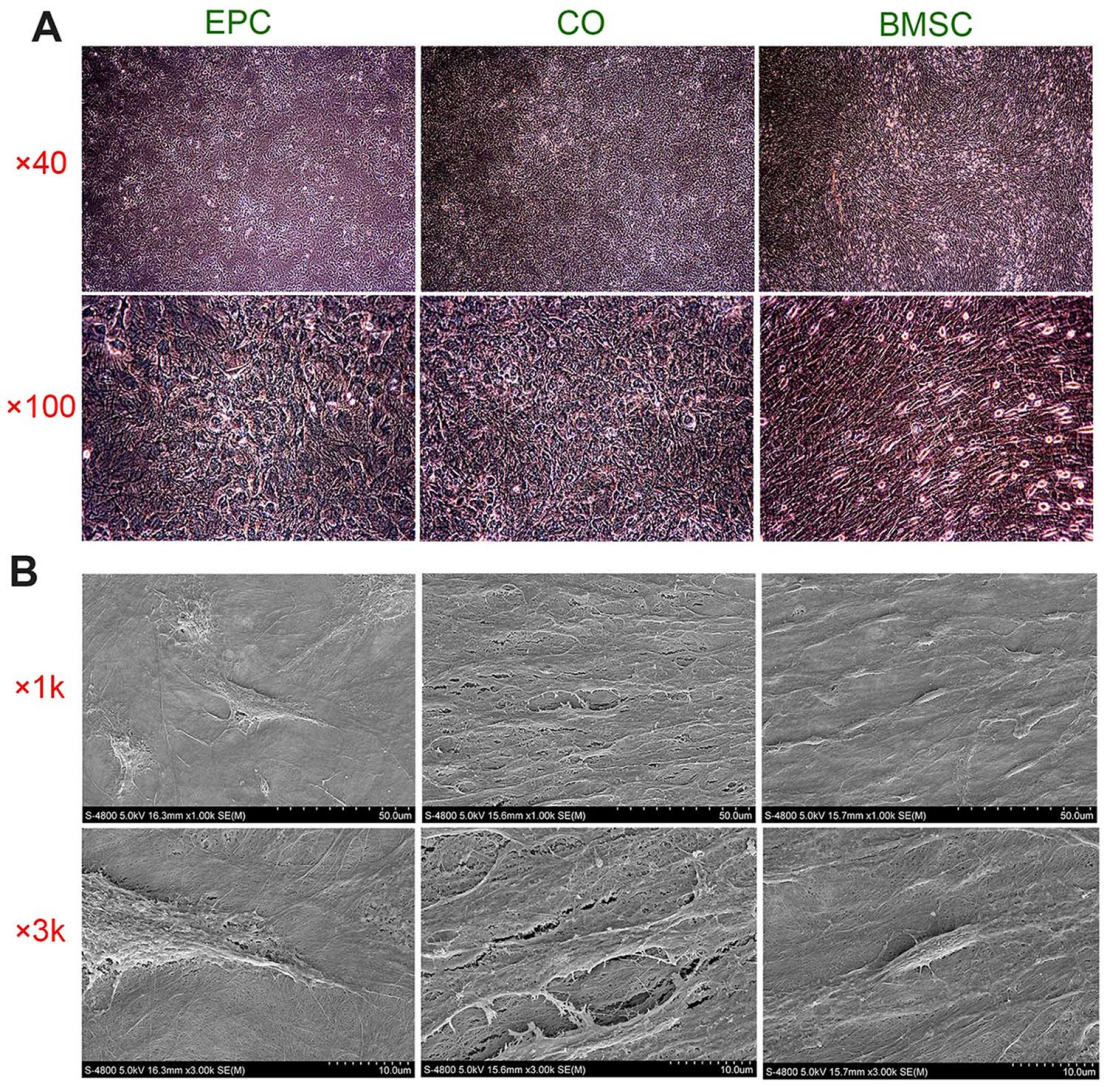
Morphology of cell sheet. (**A**) Representative microscopic view of cell sheet morphology after 8 days of induction. (**B**) Representative SEM images of the cell sheets attached to Ti discs.

BMSCs, EPCs or co-cultured cells (CO, EPC: BMSC = 10:1) were incubated in cell sheet-inducing medium to make cell sheets. The structure of cell sheets was observed under light microscopy (Fig. 2A). All of the three kinds of cell sheets were composed of layers of cells with rich ECM deposition. However, the cell morphologies of cell sheets were different. Cells of EPC sheets were round, triangular or irregular and showed random arrangement. Cells of BMSC sheets were spindle-shaped and showed a swirled pattern. Both cell morphologies of EPC sheets and BMSC sheets were observed in the co-cultured cell sheets. Cell sheets were wrapped around titanium (Ti) discs to make cell sheet-implant complexes. The scanning electron microscope examination of cell sheet-implant complexes showed ECM deposition on the surface of titanium discs, which was composed of crosslinked networks of collagen fibers and dense of cells (Fig. 2B). The shapes and arrangement patterns of cells in each group were in consistent with what were observed under light microscopy.

### *In vitro* osteogenic differentiation of cell sheet-implant complexes

To analyze the osteogenic differentiation, cell sheet-complexes of BMSC, EPC and CO group were incubated with osteogenesis-inducing medium for 5 days and tested for alkaline phosphatase (ALP) production, ECM mineralization as well as gene and protein expression.

**Figure 3:**
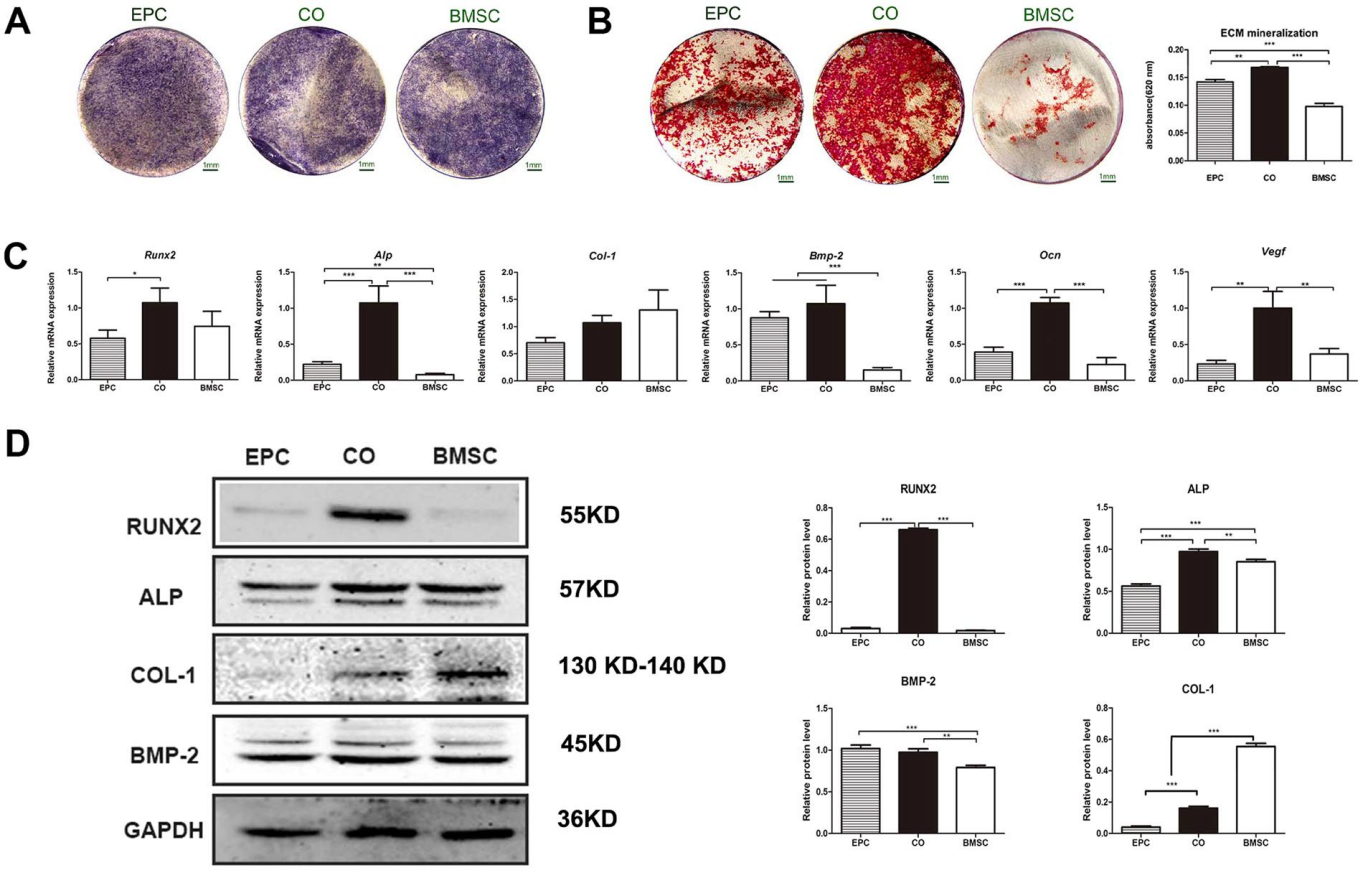
(**A**) ALP staining. (**B**) ECM mineralization and the quantitative colorimetric results of ECM mineralization. (**C**) Gene expression of *Runx2*, *Alp*, *Col-1*, *Bmp-2*, *Ocn* and *Vegf*. (**D**) Western blot analysis of RUNX2, ALP, COL-1, BMP-2 and GAPDH. The graphs show the semi-quantitative analysis of the results relative to GAPDH. (^*^p < 0.05, ^**^p < 0.01, ^***^p < 0.001).

After 5 days of osteogenic induction, all of the complexes generated certain amounts of ALP, while the production of ALP was higher in the CO group and the BMSC group than in the EPC group, as indicated by the density and area of ALP staining (Fig. 3A). The ECM mineralization was detected by Alizarin Red S. The CO group showed the highest degree of mineralization, which was revealed by the mineralized nodules and was further proved by the semi-quantitative results (Fig. 3B).

The expression of osteogenesis-related and vasculogenesis-related genes were examined by real-time PCR (Fig. 3C). The expression of *Alp, Ocn* and *Vegf* were highest in the CO group. The expression of *Runx2* was higher in the CO group and the BMSC group than the EPC group. Besides, the expression of *Bmp-2* was higher in the CO group and the EPC group than in the BMSC group while the expression of *Col-1* was relatively higher in the BMSC group without statistical significance.

The protein expression of osteogenic markers was assessed by western blot (Fig. 3D). The expression of RUNX2 and ALP were highest in the CO group. The expression of BMP-2 was higher in the CO group and the EPC group than in the BMSC group. Besides, the expression of COL-1 was highest in the BMSC group.

### Subcutaneous osteogenesis of cell sheet-implant complexes

**Figure 4:**
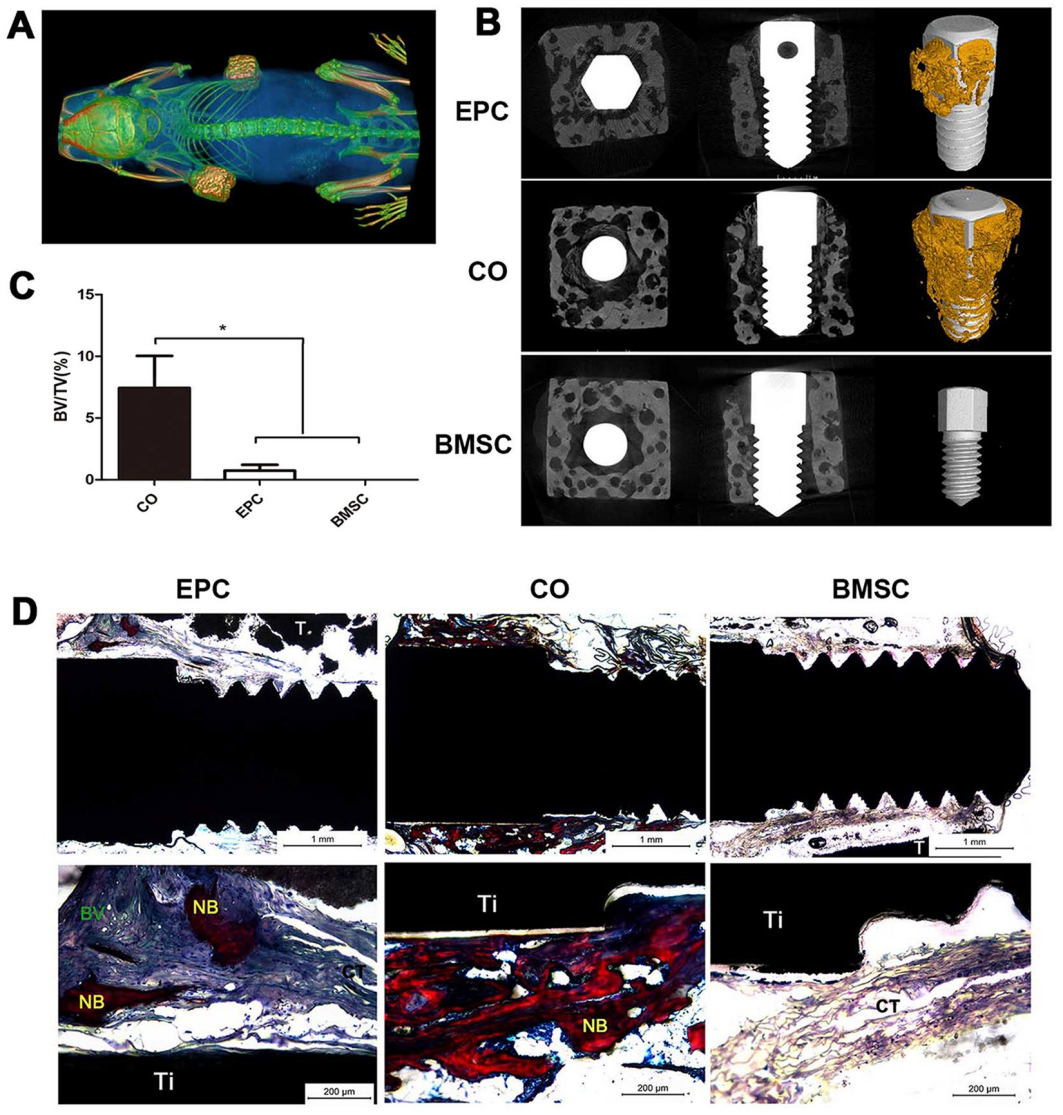
Subcutaneous osteogenesis of cell sheet-implant complexes. (**A**) Cell sheet-implant complexes transplanted into nude mice after 8 weeks. (**B**) Three-dimensional reconstruction of the EPC, CO and BMSC group. (**C**) The graph shows bone volume to total volume ration (BV/TV). (**D**) Van Gieson staining images of ectopic bone formation after 8 weeks of subcutaneous implantation. New bone formation (NB), connective tissue (CT), blood vessel (BV), implant (Ti), β-TCP (T).

Implants were wrapped with cell sheets and put into β-TCP cubes for the test of ectopic bone formation. The complexes were analyzed by micro-CT 8 weeks after transplanted subcutaneously into nude mice. The CO group exhibited obvious mineralization, as indicated by the three-dimensional stereoscopic pictures (Fig. 4B). Mineralization was also observed in the EPC group, but was significantly less than the CO group. No detectable mineralization could be found in the BMSC group. Besides, the CO group displayed the highest bone volume/total volume (BV/TV) among the three groups (Fig. 4C).

For histological analysis of new bone formation, hard tissue slices were made from the complexes, stained with van Gieson and evaluated by light microscopy (Fig. 4D). The CO group exhibited obvious newly formed bone with few fibrous connective tissues. The bone formed in the EPC group was much less than the CO group. Besides, blood vessel networks were also observed in the EPC group while only fibrous tissues could be found in the BMSC group.

### The osseointegration of different cell sheet-complexes in irradiated bone

Cell sheet-complexes of BMSC, EPC, CO group and Ti group (implants without cell sheet) were inserted in irradiated rat tibias and tested for osseointegration 8 weeks later.

**Figure 5:**
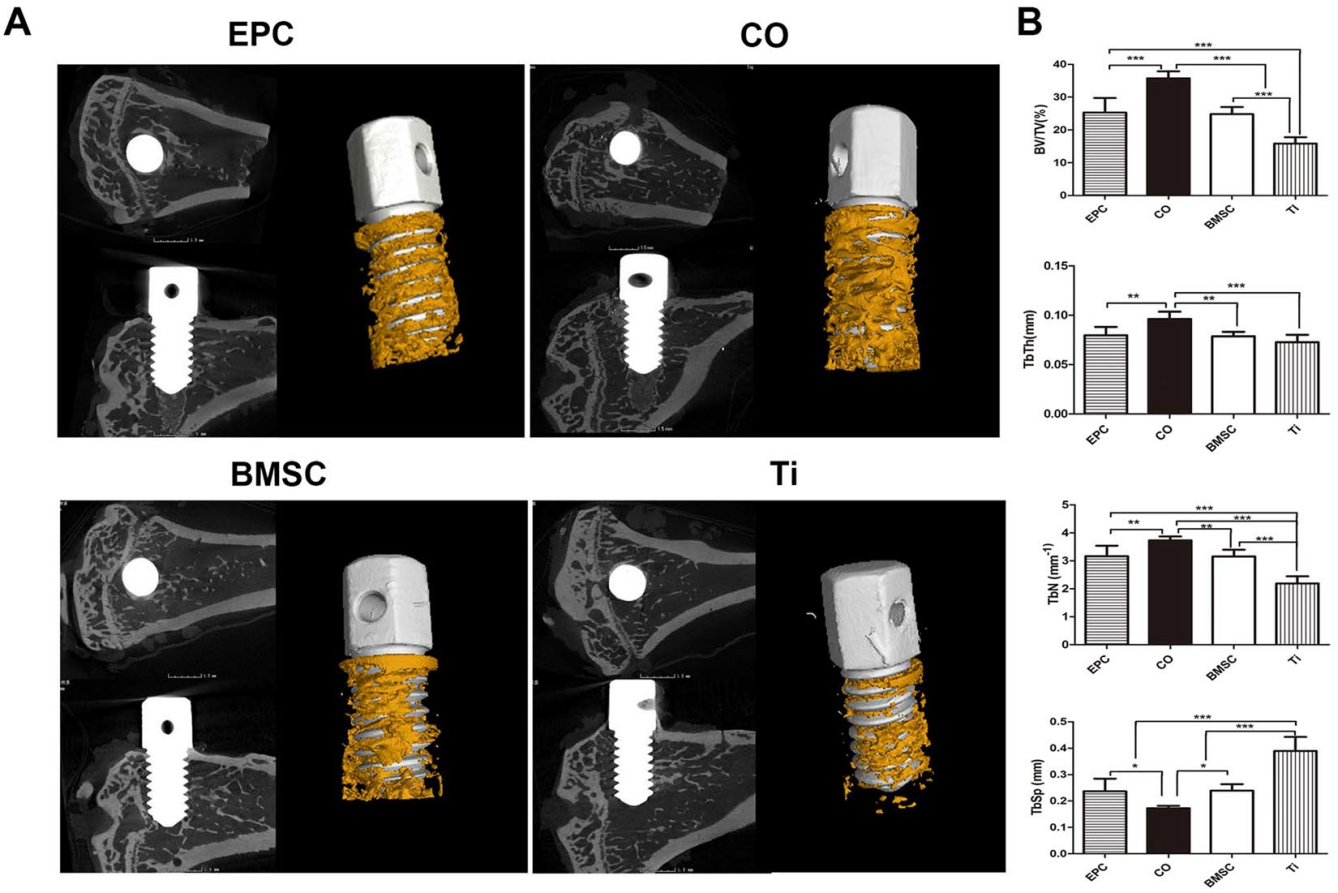
Micro-CT evaluation of bone osseointegration at 8 weeks after implantation. (**A**) 2D and 3D reconstructed images of the morphology of bone formed around implants. (**B**) Morphometric analysis of the BV/TV, Tb.N, Tb.Th and Tb.Sp. (^*^p < 0.05, ^**^p < 0.01, ^***^p < 0.001).

The structure of trabecular bone around implants was evaluated by reconstructed Micro-CT images (Fig. 5). More new bone was formed in the CO group than the other groups. The bone in the CO group displayed higher BV/TV, trabecular thickness (Tb.Th), trabecular number (Tb.N) and lower trabecular separation (Tb.Sp) than that in other groups. Both of the EPC and the BMSC group showed enhanced bone formation compared to the Ti group.

**Figure 6:**
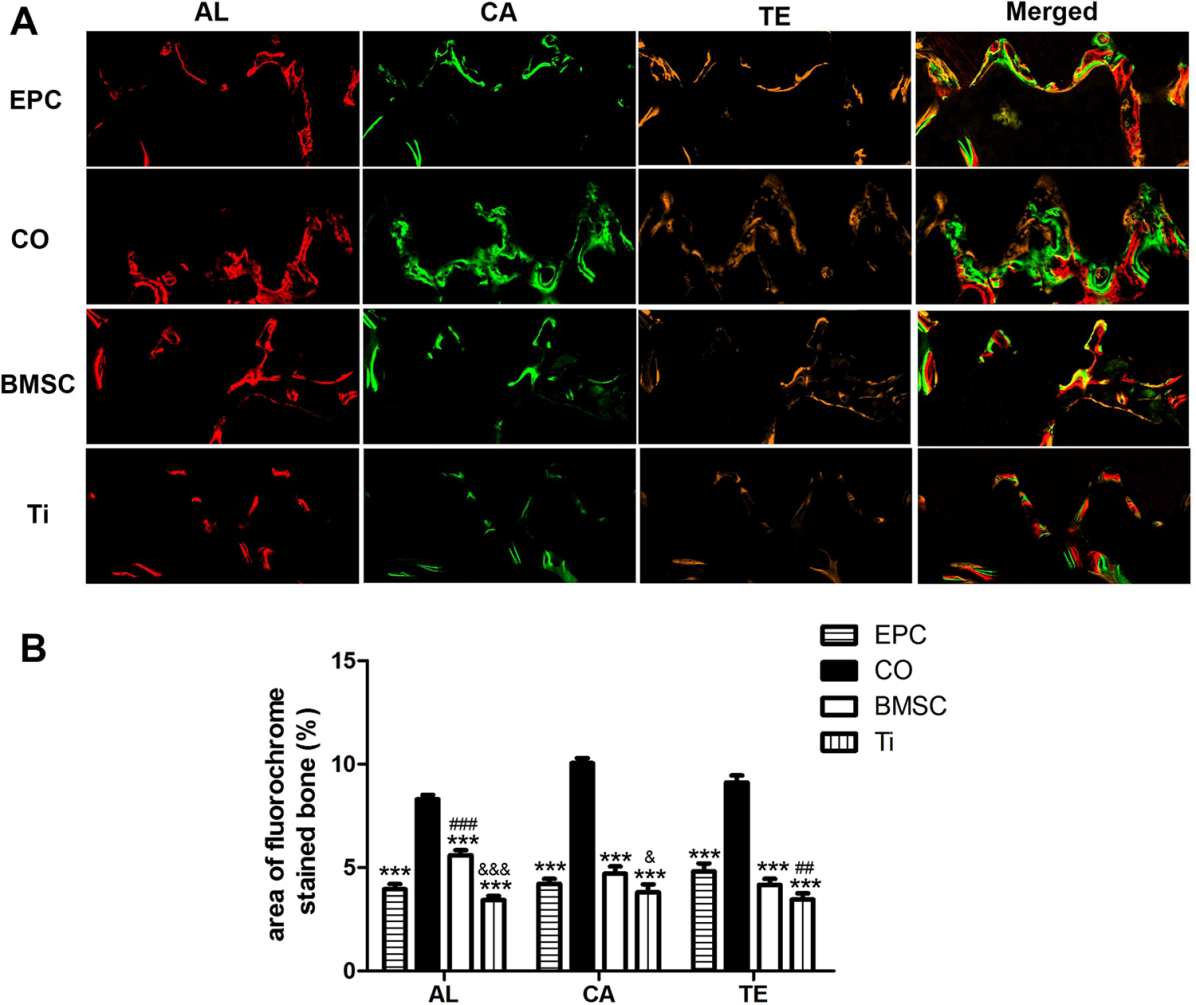
Sequential fluorescent labeling of bone formation and mineralization. (**A**) Red, green and yellow represent labels of Alizarin Red S (AL), Calcein (CA) and Tetracycline Hydrochloride (TE) respectively for each group and merged images of the three fluorochromes for the same group (Original magnification x100). (**B**) The graph shows the percentage of the three fluorochromes area in each group. (^***^p < 0.001*vs* CO, ^##^p < 0.01, ^###^p < 0.001*vs* EPC, ^&^p < 0.05, ^&&&^p < 0.001 *vs* BMSC).

Sequential fluorescent labeling was used to measure the bone mineralization and deposition (Fig. 6). The CO group showed the highest percentage of fluorescent labeling at each time point. Both of the EPC group and the BMSC group showed a slightly higher percentage of fluorescent labeling compared to the Ti group. Taken together, the CO group is more beneficial for new bone formation and mineralization than the other groups.

**Figure 7:**
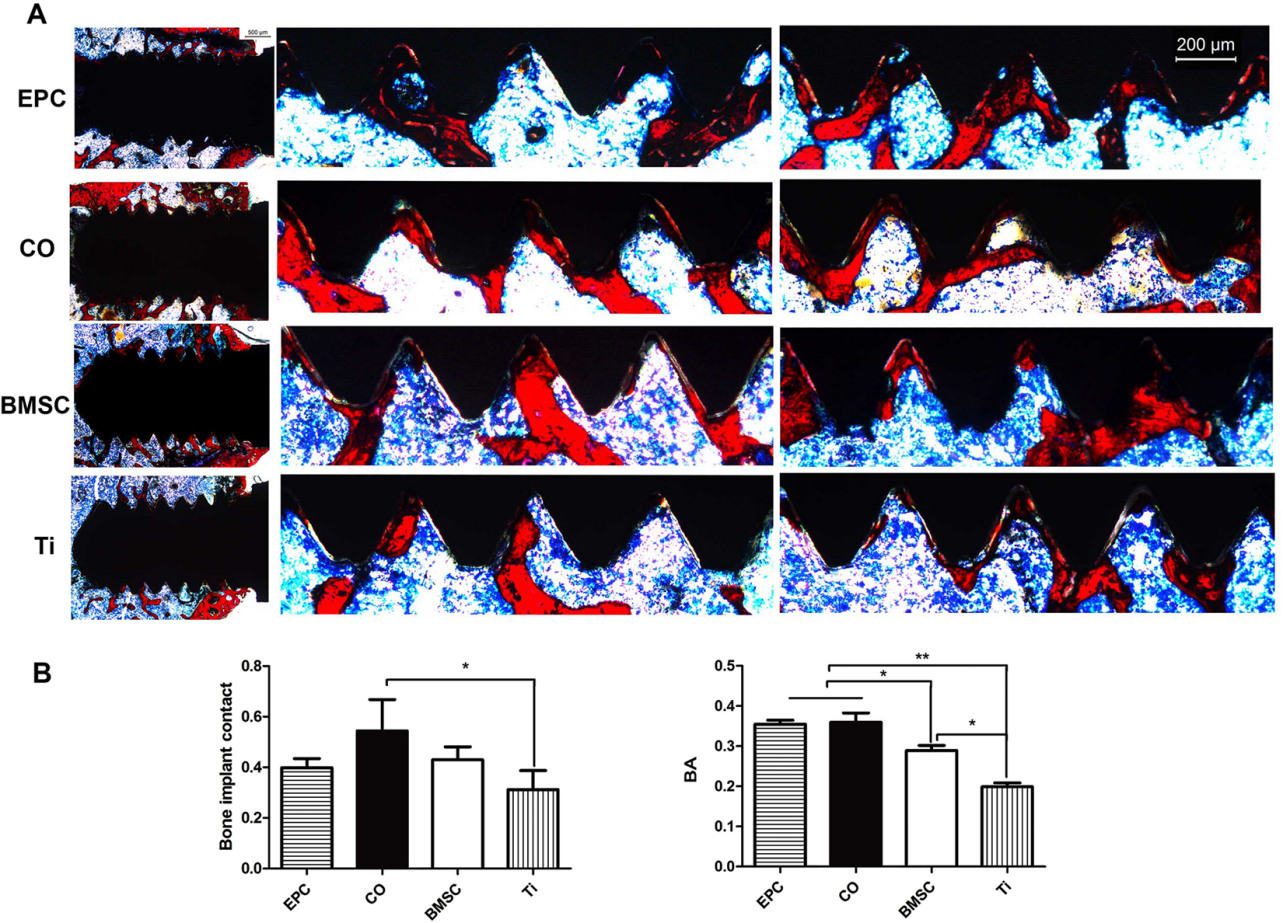
Histological analysis of newly formed bone around implants. (**A**) The first column shows the overall image of implants of each group (Original magnification x25) and the second and third column show bone formation around the upper and lower screws (Original magnification x50). (**B**) The graphs show the BIC and BA per x50 field in histological sections. (^*^p < 0.05, ^**^p < 0.01).

To further investigate the osseointegration, hard tissue sections with Van Gieson’s staining were observed under light microscopy (Fig. 7). The bone formed around implants in the Ti group was much less than that in the other three groups, demonstrating that cell sheets could enhance osseointegration. Both of the CO group and the EPC group showed the largest peri-implant bone formation area (BA). The BMSC group also showed larger BA than the Ti group. The CO group displayed the highest bone-implant contact (BIC). Besides, the bone structure in the CO group was more continuous than the other groups.

**Figure 8:**
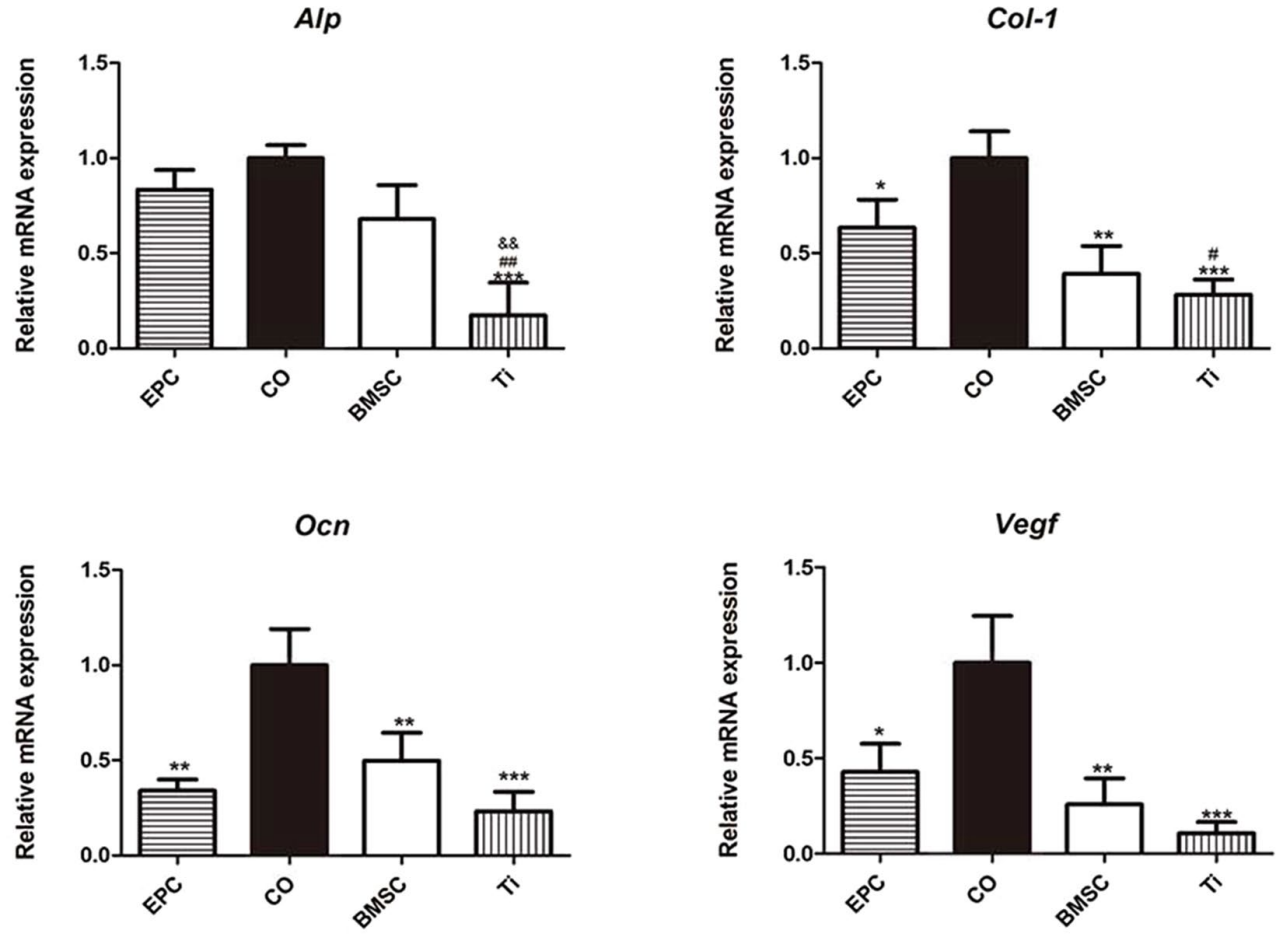
Gene expression of the bone fragments around implants in each group. ^*^p < 0.05, ^**^p < 0.01,^***^p < 0.001*vs* CO, ^#^ *p* < 0.05, ^##^p < 0.01*vs* EPC, ^&&^p < 0.01 *vs* BMSC.

The expression of osteogenesis-related and angiogenesis-related genes was tested in peri-implant bone. The CO group displayed the highest expression level of *Alp*, *Col-1*, *Ocn* and *Vegf*. Besides, the EPC group expressed a higher level of *Vegf* than the BMSC group and the Ti group. (Fig. 8)

## Discussion

MSCs and EPCs have been demonstrated as attractive cell sources for tissue engineering applications^16^. In this research, we used cell sheets of co-cultured BMSCs and EPCs to improve osseointegration in irradiated bone. At the moment, there is no specific marker that allows isolation of a pure population of mesenchymal stem cells or EPCs from bone marrow. The common procedure allows the isolation of a heterogeneous population that contain stem cells, progenitors and differentiated cells^17^. Thus, the identity of the cell populations is pivotal for their potential clinical applications. The cultured cells showed typical characteristic of BMSCs and EPCs as shown in Fig. 1. Besides, real-time PCR of the third passage of BMSCs and EPCs were also performed (Supplementary Fig. S1D). EPCs had significantly higher expression levels of *Nos3* and *Vwf* than BMSCs, which are endothelial markers. Besides, EPCs also expressed a higher level of *Bmp2*. BMPs are crucial factors for regulation of angiogenesis and bone formation^18^. BMPs, including BMP2, can also be used as a marker of the late EPCs as well as a marker for angiogenesis^19^. What’ more, senescence is a key point that must be considered when using cells for transplantation purposes^20^. Despite that cells at the third passage was not greatly affected by senescence, we evaluated the percentage of senescent cells of both EPCs and BMSCs (Supplementary Fig. S1A,B). 2.56 ± 0.73% EPCs and 2.13 ± 0.58% BMSCs stained with senescence-associated β-galactosidase at P3.

An intimate functional relationship exists between vascular endothelium cells and osteoblastic cells during osteogenesis^21^. In order to improve bone tissue engineering approaches, studies are concentrated on the co-culture of osteogenic and endothelial lineages^22^, which interact with each other to regulate differentiation. EPC holds great promise in regenerative medicine because it has higher potential of proliferation and survival than mature endothelial cells^23^. Studies demonstrated that co-cultured EPCs and BMSCs could enhance bone regeneration^14, 24, 25^. However, no consensus on the condition for co-culture has been achieved^26^. In order to determine the best ratio of co-culture for bone regeneration, we co-cultured EPCs and BMSCs at ratios of 1:0, 10:1, 5:1, 1:1, 1:5, 1:10 and 0:1. The co-culture ratio of 10:1 showed the optimal level of osteogenic related gene expression (Supplementary Fig. S2). This ratio was reported to be able to ensure the presence of both cell types in the co-culture system^27^. The upregulation of osteoblastic markers indicated that the co-cultured cells had enhanced osteogenic differentiation. This is in accordance with other studies^28, 29^. Besides, our study showed that EPCs had some osteogenic potential, which has been explored by other studies^23, 30^.

Many researchers have designed strategies to enhance bone formation based on simultaneous culture of EPCs and BMSCs^14, 26, 29, 31–34^. The co-culture systems usually use conventional tissue engineering methods of seeding cells into scaffolds. However, this method often leads to a significant loss of cells. Besides, the use of proteolytic enzymes to prepare cell suspensions can result in cell damage and loss of differentiated phenotypes^35^. Cell sheet engineering harvests cultured cells as intact sheets, which causes minimal cell loss and can be directly transplanted to tissue beds ^36^. Therefore, we used cell sheet engineering in this study. BMSC cell sheets have been used in various studies^37^, but application of EPC cell sheets and cell sheets of co-cultured EPCs and BMSCs are rarely reported. We found that EPCs and co-cultured cells could survive in the medium containing Vc and form cell sheets. These two kinds of cell sheets formed faster than BMSC cell sheets and were thicker when we harvested them for later use.

In a previous study, we demonstrated that it is possible to promote osseous healing by BMSC-Implant complex. Compared with traditional methods of altering surface characteristics such as roughness and chemistry, BMSC-Implant complex could accelerate bone healing by providing extracellular matrix^11^. It is also a more direct and economical way to enhance tissue response compared with biochemical surface modification. BMSC is known to be able to adhere to the titanium surface. BMSC cell sheet also could attach to the titanium implant as proved by our previous work. A recent study showed that there is interaction between EPC and titanium implant surfaces^38^. To explore whether EPC or co-cultured cell sheets could also promote the osseointegration of titanium implant, we firstly assessed their attachment to titanium discs. Cell sheets of all the groups formed stable attachment, as indicated by cells with cell-to-cell junctions and crosslinked networks of collagen fiber on the surface of titanium discs. The co-cultured group showed higher densities of cells and collagen fibers, suggesting that cells of this group have a higher proliferation rate and a higher amount of ECM deposition.

Osteogenic differentiation of cell sheet-implant complexes were analyzed *in vitro* and *in vivo*. In general, the co-cultured group demonstrated enhanced osteogenic differentiation which is in accordance with most of the studies^[32,39^. The co-cultured group showed improved ECM mineralization and a higher expression of RUNX2, ALP and BMP-2 at both mRNA and protein levels, indicating an accelerated osteogenic differentiation process. Besides, the gene expression of *Vegf*, which can be secreted by both BMSC and EPC^40^ and plays a central role in osteo-endothelial communication in co-culture models^22^, was significantly up-regulated in the co-cultured group. VEGF is important for neovascularization and bone healing^38^. It promotes mobilization, recruitment, and differentiation of EPCs. It also influences the recruitment and differentiation of osteogenic cells. Improved osteogenic differentiation of co-cultured group was further confirmed by ectopic bone formation. In this test, β-TCP cubes were used to mimic the bone structure around implants. Abundant bone formation was observed in the co-cultured group by Micro CT scanning and histological analysis. These results provide a good basis for studies to detect their function in osseointegration.

Implants in irradiated bone have 2–3 times higher rates of failure^41^, which makes maxillofacial prosthetic rehabilitation based on implant retention difficult. Radiation impairs osseointegration due to its adverse effect on the physiology of bone. Local radiation leads to deprivation of osteocyte and osteoblast^42^. Differentiation of the surviving MSCs and osteoprecusor into osteoblast is also inhibited^42^. Decreased tissue perfusion leads to further deleterious outcomes^43^. A study showed that irradiated cultures of osteoblast like cells resulted in diminished production of VEGF^44^. In our study, improved osseointegration in irradiated rat tibias was obtained in the co-cultured group. More new bone formed around implants in the co-cultured group, as revealed by Micro-CT analysis. Besides, the osseointegration process was faster in the co-cultured group, as demonstrated by fluorescent labeling of bone mineralization and deposition. Histological examination demonstrated that the bone structure was more continuous and homogeneous in the co-cultured group. Although fibrous tissues was found around implants in the study of ectopic bone formation, no fibers were present between the irradiated bone and the implant surface. This might be due to the osteoinduction of bone tissue. Furthermore, cell sheets of co-cultured EPCs and BMSCs up-regulated a panel of genes of osteoblast markers and *Vegf* in peri-implant bone. These data proved that cell sheets of co-cultured EPCs and BMSCs can promote osseointegration in irradiated bone. The possible mechanism for the enhancement may be that EPCs and BMSCs could make up for the hypocellular state and the high expression of *Vegf* could improve the angiogenesis and osteogenesis.

BMSC-implant complexes have been used in diabetic rats^45^. However, there are concerns about the potential dislocation of the cell sheet toward the end of the implant at the moment of its placement. This phenomenon was observed in this study. In order to leave enough cell sheet membrane around the whole implant, we put a piece of cell sheet in the hole before the placement of the implant. Parts of the membrane moved toward the top end of the implant, but much less than what we put in the hole and wrapped around the implant. The feasibility of this method was further comfirmed by the enhanced peri-implant bone formation and osseointegration in the irradiated bone. The mechanism of how cell sheets of co-cultured EPCs and BMSCs improved the osseointegration needs to be further explored.

## Conclusion

It is a novel approach to enhance implant performance by cell sheet engineering. Cell sheets of co-cultured EPCs and BMSCs showed excellent osteogenic differentiation around titanium materials *in vitro* and enhanced ectopic bone formation. The co-cultured cell sheets enhanced osseointegration in irradiated rat tibias. The up-regulation of osteogenesis-related gene expression in peri-implant bone indicated that bone viability was enhanced, which is of critical importance to bone healing around implants. Our findings suggest that cell sheets of co-cultured EPCs and BMSCs have the potential to improve osseointegration in irradiated bone.

## Materials and Methods

### Ethics statement

All animal experimental protocols were reviewed and approved by the Animal Care Committee of Fourth Military Medical University (Permit Number: 15007), and all experiments were performed in accordance with the relevant guidelines and regulations. Sprague-Dawley of 2 weeks-old were used for the isolation of BMSCs and EPCs. Five female nude mice of 6 weeks-old were used in the subcutaneous ectopic osteogenesis experiment. 18 male Sprague-Dawley rats were used in implant experiment. Animals were kept in specific pathogen-free conditions (SPF) at 26 °C with a 12-hour light/dark cycle. They were given a standard pellet rodent diet and water. All surgeries were performed under sodium pentobarbital anesthesia, and all efforts were made to minimize suffering.

### Culture and characterization of BMSCs

Rat BMSCs were isolated and cultured as reported^46^. Cells of the third passage were tested for osteogenic, adipogenic and chondrogenic differentiation and cell surface markers of CD29, CD44, CD90, CD31 and CD34. Detailed information can be found in Supplementary Methods.

### Culture and characterization of EPCs

Rat bone marrow derived EPCs were isolated and cultured as reported^47^. EPCs of the third passage were tested for cell surface markers of CD31, CD34, CD144 and VEGFR2. The ability of capillary tube formation and uptake of Dil-Ac-LDL and FITC-UEA-1 was also tested. EPCs were observed by Transmission electron microscopy for Weible-Palade bodies. Detailed information can be found in Supplementary Methods.

### Fabrication of cell sheet-implant complex

The third passage of BMSCs, EPCs or a mixture of them (EPC:BMSC = 10:1, according to the preliminary test) were seeded in 6-well plates at the density of 2.75 × 10^5^ cells/well. The cell culture medium was shifted to cell sheet-inducing medium (α-MEM supplemented with 10% FBS, 50 mg/ml Vc and 1% penicillin and streptomycin) after the cells reached 95% confluence. Cell sheets were formed after 8 days of culture. After detached from the plates, one layer of cell sheet was wrapped around a titanium (Ti) disc or a Ti implant (99.99% pure; Zhong Bang Corporation, China) (Supplementary Fig. S3). Ti discs (L:2 mm; Φ:10 mm, smooth surface) were used for *in vitro* studies while Ti implants (L:6 mm; Φ:1.9 mm, smooth surface) were used for *in vivo* studies. To observe cell sheets’ attachment to Ti discs, the complexes were cultured for 48 h and then fixed in 4% paraformaldehyde, dehydrated and sputter coated with gold. The samples were examined by a scanning electron microscope (S-4800, HITACHI, Japan).

### *In vitro* osteogenesis of different cell sheet-implant complexes

For *in vitro* osteogenic differentiation analysis, cell sheet-complexes of BMSC, EPC or CO group were incubated in osteogenesis-inducing medium for 5 days. ALP production, ECM mineralization as well as osteogenesis-related gene and protein expression were tested. Detailed information can be found in Supplementary Methods.

### Subcutaneous osteogenesis of different cell sheet-complexes

To assess ectopic osteogenesis of cell sheet-complexes, implants were wrapped with cell sheets and put into the cylindrically shaped space (L:5 mm; Φ:2.5 mm) in β-TCP cubes (5 × 5 × 5 mm, porosity 60%, pore size 380 μm; Wuhan Huawei Biomaterials and Engineering, China) (Supplementary fig. S3B). Nude mice were intraperitoneally injected with pentobarbital sodium solution (1% w/w) (40 mg/kg) to induce anesthesia. Then, cell sheet-implant complexes were subcutaneously transplanted into the backs of nude mice (n = 3). 8 weeks after implantation, the mice were sacrificed and samples were harvested for micro-CT analysis and hard tissue slices examination.

### Test of Micro-CT

Nude mice were scanned by Micro-CT (Inveon CT, Siemens, Germany) with a scanning resolution of 22 µm. Afterwards, the cell-sheet complexes were harvested, fixed in 4% paraformaldehyde and scanned by Micro-CT (Y.XLONY.Cheetah, Germany) with a scanning resolution of 13 µm. VG StudioMAX (Volume Graphics, Germany) was used for 3D reconstructing image and data analysis. Gray value of 550 was chosen to distinguish the bone and β-TCP and 1500 was chosen to distinguish the bone and Ti implant. Bone volume/total volume (BV/TV) was calculated.

### Examination of hard tissue slices

After fixation, cell sheet implant-complexes were dehydrated in ascending concentrations of ethanol, embedded in methyl methacrylate and cut into sections with hard tissue slicer (LEICA SP1600, Germany).These sections were 50 μm and stained with van Gieson. Histological analyses were performed using the light microscope (Leica DMI6000, Germany).

### Osseointegration of different cell sheet-complexes in irradiated bone

#### Experimental Design

Male Sprague-Dawley rats weighing 240–270 g were used. Implant surgeries were performed 8 weeks after the rats received irradiation for their tibias. All the 18 rats with 36 implant sites were randomly allocated into the following four groups: (1) EPC (n = 9), (2) Co-cultured EPCs and BMSCs (CO, n = 9), (3) BMSC (n = 9), (4) Titanium (Ti, = 9). All animals were sacrificed 8 weeks later by intra-abdominal administration of excessive pentobarbital sodium solution and samples were harvested. Six samples of each group were used for micro-CT analysis and hard tissue slices examination and three samples for gene expression.

#### Radiation

Rats were immobilized in a specially designed thermoplastic resin rack under general anesthesia (Supplementary Fig. S4A).The tibias of each rat were subjected to a single dose of 20 Gy. The field size was 20 × 20 cm and the source-skin distance was 100 cm. A 2 cm thick lead shielding was used to protect other parts of rats from radiation (Supplementary Fig. S4B). Radiotherapy was performed with the 23EX medical linear accelerator (Varian, USA), energy 6 MeV, dose rate 4 Gy/ minute (Supplementary Fig. S4C).

#### Implant surgery

Implant surgery was performed 8 weeks after the irradiation. Rats were fixed in the supine position after being anesthetized. The surgical area was shaved and cleaned with 0.5% iodophor and 70% ethanol. An incision of about 15 mm long was made on the mesial surface of both tibias to expose the tibia metaphysis. A hole of 1.8 mm was drilled 2–3 mm distal to the epiphysis line with sterile saline irrigation (Supplementary Fig. S5A). After a layer of cell sheet were put inside the hole, an implant wrapped with a piece of cell sheet of the same group were screwed in (Supplementary Fig. S5B-D). Afterwards, muscle tissue and skin were sutured separately (Supplementary Fig. S5E and F). Antibiotics were administered for 3 consecutive days post-surgery.

#### Sequential fluorescent labeling

To assess the new bone formation and mineralization around implants, different fluorochromes were injected intramuscularly. Alizarin Red S (Sigma, USA, 30 mg/kg) injections were performed at 2 and 3 weeks post-operation. Calcein (Sigma, USA, 20 mg/kg) injections were performed at 4 and 5 weeks post-operation. Tetracycline Hydrochloride (Sigma, USA, 20 mg/kg) injections were performed at 6 and 7 weeks post-operation.

#### Gene expression analysis

Bone fragments around implants (within1.5 mm from the surface) for each group were immediately frozen in liquid nitrogen and grinded. The total RNA was extracted with Trizol reagent (Invitrogen, USA). The following procedures were the same as mentioned above.

#### Test of Micro-CT

Micro-CT scanning procedure was the same as mentioned above. For the evaluation of bone formation around implants, the region of interest (ROI) was defined as the region left after subtracting the implant from a cylindrical region (L:3.2 mm; Φ:2.4 mm). Bone volume/total volume (BV/TV), Trabecular Number (Tb.N), Trabecular Thickness (Tb.Th) and Trabecular Separation (Tb.Sp) were calculated and recorded.

#### Examination of hard tissue slices

The undecalcified sections were prepared as mentioned above. To analyze mineralization in the bone around implants, the fluorescent labeling was observed under Laser Scanning Confocal Microscope (OLYMPUSFV1000, Japan) as reported before^48^. After the sections were stained with Van Gieson, bone-implant contact (BIC) and peri-implant bone area (BA, total area of bone in the reference area/total reference area) were quantified for four threads in a row using a computer-based image analysis system (Image Pro 5.0).

### Statistical analysis

Results were expressed as mean ± standard deviation. Data were analyzed by one-way ANOVA. If differences were significant, a Tukey post-hoc test was used to perform pair-wise comparisons. SPSS 15.0 software was used and statistical significance was considered when p < 0.05.

## Electronic supplementary material

Supplementary Information
