# Supplementary material for: Cell Sheets of Co-cultured Endothelial Progenitor Cells and Mesenchymal Stromal Cells Promote Osseointegration in Irradiated Rat Bone
Source: Sci Rep. 2017 Jun 8;7:3038. doi: 10.1038/s41598-017-03366-9 (PMC5465198; doi:10.1038/s41598-017-03366-9)
Supplement: Supplementary file 1 — Supplementary Information [file 41598_2017_3366_MOESM1_ESM.pdf]

Cell Sheets of Co-cultured Endothelial Progenitor Cells and Mesenchymal Stromal Cells Promote Osseointegration in Irradiated Rat Bone

Huan Liu[1, ¶], WeiZhou[1,¶], NanRen[1], Zhihong Feng[1] , Dong Yan[1], Shizhu Bai[1], Yang Jiao[2], Zhongshan Wang[1], Yimin Zhao[1,*].

1. State Key Laboratory of Military Stomatology & National Clinical Research Center for Oral Diseases & Shaanxi Key Laboratory of Stomatology, Department of Prosthodontics, School of Stomatology, The Fourth Military Medical University, Xi'an, China

2. Department of Stomatology, PLA Army General Hospital, 100700，Beijing, China

* Corresponding author.

E-mail address: zhaoymdentist@126.com

[¶]These authors contribute equally to this work.

Supplementary Methods

Culture and characterization of BMSCs

Isolation and culture of BMSCs

Rat BMSCs were isolated and cultured as it reported[1]. Briefly, tibias and femurs were removed after the rats were euthanized. Bone marrow was flushed out with α-minimum essential medium (α-MEM, Gibco, USA) supplemented with 10% fetal bovine serum (FBS, Hangzhou Sijiqing Biological Engineering Materials Co., Ltd. China) and 1% penicillin and streptomycin. Upon reaching 80% confluence, adherent cells were trypsinized and sub-cultured for further experiments.

Differentiation assays of BMSCs

For osteogenesis, cells were incubated in osteogenesis-inducing medium (10 mM β-glycerolphosphate, 50 μg/ml Vc and 0.1mM dexamethasone, all from Sigma–Aldrich) for three weeks. The cells were stained with Alizarin Red S (Sigma–Aldrich, USA) after fixation. For adipogenesis, the cultured cells were incubated in adipogenic differentiation medium (50mM indomethacin, 100nM dexamethasone, 10 mM insulin, 0.5mM methylisobutylxanthine, all from Sigma–Aldrich) for two weeks. The cells stained with Oil Red O (Sigma–Aldrich) after fixation. For chondrogenesis, cells were centrifuged to form a pelleted micromass and were incubated in chondrogenic differentiation medium (Cyagen, China, RASMX-90042) for 28 days. Pellets were formalin fixed and paraffin embedded for Alcian blue stain (Cyagen, China).

Flow cytometry analysis

5×10^5 cells were used for each test. The cells were incubated for 30 min with

fluorescent isothiocyanate (FITC)-conjugated mouse anti–rat CD31 (Abcam, ab33858), FITC-conjugated mouse anti–rat CD44 (Abcam, ab33900), FITC-conjugated mouse anti–rat CD90 (eBioscience, 11-0900), Rabbit anti-CD34 (Abcam, ab81289) or biotin-conjugated Hamster Anti-Rat CD29 (Abcam, USA) at 4 ℃ for 30 minutes. The cells incubated with anti CD31 were incubated with donkey anti-rabbit IgG-FITC (Santa Cruz Biotechnology, sc-2090) afterwards. The cells incubated with anti-CD29 were incubated with PE/Cy5 Streptavidin (Biolegend, 405205) afterwards. Cells without incubating with antibody were served as controls. Beckman Coulter Fc500 (USA) was used for the test.

Culture and characterization of EPCs

Isolation and culture of EPCs

Rat EPCs were isolated and cultured as reported before[2]. The bone marrow suspension was fractionated by density gradient centrifugation (Histopaque-1083, Sigma, USA) for 30 min at 400g. The mononuclear cells were washed three times with PBS at 250g for 10 min. The cell pellet was then suspended in EBM-2 medium with EGM-2 MV SingleQuots (Lonza, USA) and plated on fibronectin-coated culture dishes. After 48h the non-adherent cell population was aspirated and transferred to new fibronectin-coated dishes. The culture medium was changed every 2 days and cultured for a total of 13 days. The adherent cells were subcultured for further experiments.

Flow cytometry analysis

EPCs were tested for CD31 (Abcam, ab33858), CD34 (Abcam, ab81289), CD144 (Abcam, ab166715) and VEGFR2 (Abcam, ab9530). Methods were as mentioned

above.

Dil-Ac-LDL and FITC-UEA -1 uptake

Cells were cultured for 24h and incubated with 40 μg/ml dil acetylated low density lipoprotein (DiI-Ac-LDL, Molecular Probes, USA) for 4 h at 37 ℃ and 5% CO2. After washing three times with PBS, the cells were incubated with 10ug/mL fluorescein isothiocyanate conjugated Ulex europaeus agglutinin (FITC-UEA-1, Sigma-Aldrich, USA) for 2h. Cells were then stained with DAPI (Bioworld Technology, USA) and observed under Laser Scanning Confocal Microscope (OLYMPUSFV1000, Japan)

Transmission electron microscopy

10^6 cells were collected and fixed in 3% glutaraldehyde for 2h at room temperature. The samples were post-fixed in 1% OsO4 for 2h and dehydrated in ascending concentrations of acetones followed by embedding in Epon resin. The prepared tissue was cut at 50nm and stained with uranyl acetate and lead citrate, and then observed under FEI Tecnai G2 transmission electron microscope (USA) and photographed.

Capillary tube formation assay in Matrigel

200μl of Matrigel was laid into the wells of a 24-well plate and incubated at 37 ℃ for 45 minutes. 5×10^4 EPCs in 500ul EGM-2MV were added into each well. Capillary tube formation on Matrigel (BD, USA) was observed after 4 hours of incubation.

Senescence-associated β-galactosidase staining

Senescence of cultivated BMSCs and EPCs at passages 3 were studied using senescence-associated β-Galactosidase Staining Kit (Beyotime, China) according to the manufacturer's protocol. At the end of staining procedure, five pictures were taken from

random areas of each culture. The percentage of senescent cells was calculated (number of cells with intracellular blue deposits/ total number of cells ×100%).

In vitro osteogenesis of different cell sheet-complexes

ALP production

Cell sheet-complexes were washed with PBS and fixed in 4% paraformaldehyde for 15min, stained with the BCIP/NBT ALP color development kit (Beyotime, China) for 10minutes at room temperature, and were observed and photographed by Stereo Microscope.

ECM mineralized nodule staining

After fixation, cell sheet-complexes were stained with 1wt% Alizarin Red S for 5 minutes at room temperature, and several washes with PBS were used to remove unbound dye. The stained calcium nodules were then observed and photographed by Stereo Microscope. For quantification, the stain was dissolved with 350ul of destain solution (10% (w/v) cetylpyridinium chloride in 10 mmol sodium phosphate) for 1h in 12-well plates. The absorbance was quantitatively measured at 620nm.

Quantitative Real-time Polymerase Chain Reaction

Total RNA was isolated using TriZol (Invitrogen, USA). Total RNA was transcribed into complementary DNA (cDNA) using a PrimeScript RT reagent kit (TaKaRa, Japan). The analysis was performed on the CFX96™Real-Time PCR System with SYBR PremixExTaq™II (TaKaRa, Japan). The following components were prepared:1μlcDNA(50ng/μl), 5ul SYBR PremixEx Taq™II, 0.4μl Forward Primer(5μM), 0.4μl Reverse Primer(5μM) and H_2O was added to a final volume of

10μl. The PCR conditions were 95 ℃ for 3m followed by 40 cycles of 95 ℃ for 10s and 60 ℃ for 30s. All of the reactions were run in triplicate and were normalized to *Gapdh*. The relative gene expression was determined using the ΔΔCt method. The primers were synthesized as shown in Supplemental Table S1.

Supplemental Table S1. Primers used for Real Time RT-PCR

Gene	Forward primer sequence(5'-3')	Reverse primer sequence(5'-3')
Runx2	5' AGA CCA GCA GCA CTC CAT AT 3'	5' CTC ATC CAT TCT GCC GCT AGA 3'
Col-1	5' GCCTCCCAGAACATCACCTA3'	5' GCAGGGACTTCTTGAGGTTG 3'
Alp	5' ATG GCT CAC CTG CTT CAC G 3'	5' TCA GAA CAG GGT GCG TAG G 3'
Bmp-2	5' ATG GGT TTG TGG TGG AAG TG 3’	5' TTG GCT TGA CGC TTT TCT CG 3'
Ocn	5' AGG GCA GTA AGG TGG TGA AT 3'	5' GCA TTA ACC AAC ACG GGG TA 3'
Vegf	5' AGG AGT ACC CCG ATG AGA TA 3	5' CTT CTA CTG CCC TCC TTG TA 3'
Gapdh	5'GGCACAGTCAAGGCTGAGAATG3'	5' ATGGTGGTGAAGACGCCAGTA 3'
Nos3	5' CAG AGA GGC AAA GAA ATG GT 3'	5' AAC CTT GGC TTC TGT CAG TG 3'
Vwf	5' AGT AAC GGG AGT GAT TCA GT 3'	5' TGG GCT CCT CTC TAA GTA AA 3'

Western blot analysis

The total proteins were extracted from the cell sheet-complexes by lysing in RIPA buffer with a protease inhibitor cocktail (Sigma, USA). Protein concentration was quantified using a coomassie brilliant blue (CBB) G250 protein assay kit (Beyotime, China). The proteins were separated by SDS-PAGE and transferred to the nitrocellulose membrane. After blocked with 5% BSA for 2 h, the membranes were then incubated with primary antibodies for rat RUNX2 (Santa Cruz Biotechnology, sc-10758), ALP (Abcam, ab65834), COL-1(Abcam, ab90395), BMP2 (Abcam, ab14933) and GAPDH (Abcam, ab8245).Then the membranes were incubated for 2 h with secondary antibodies (Cowin Biotech Co., Ltd, Beijing, China). Finally, the membrane was visualized with an enhanced chemiluminescent detection system

(Amersham Biosciences, USA).The gray values of the protein bands were quantified by using Image-Pro Plus 6.0 software.

References:

1. Yan, J. et al. Non-Viral Oligonucleotide Antimir-138 Delivery to Mesenchymal Stem Cell Sheets and the Effect On Osteogenesis. *BIOMATERIALS*. 35, 7734-7749 (2014).

2. Kahler, C. M. et al. Peripheral Infusion of Rat Bone Marrow Derived Endothelial Progenitor Cells Leads to Homing in Acute Lung Injury. *Respir Res*. 8, 50 (2007).

Supplementary figures

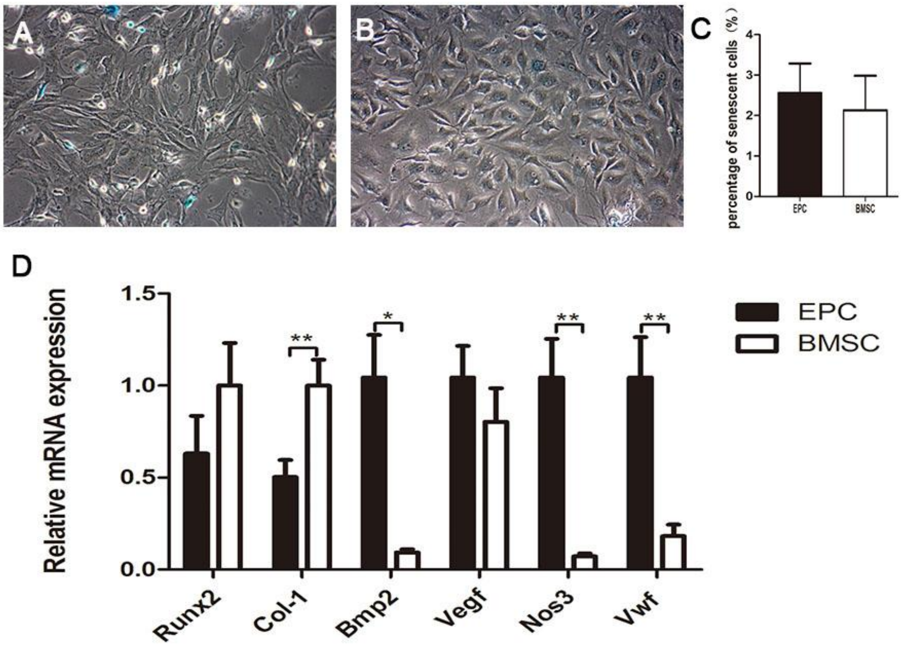

Supplementary Fig. S1. Characterization of BMSCs and EPCs of P3 A: β-galactosidase staining of BMSCs. B: β-galactosidase staining of EPCs. C: The graph shows the the percentage of senescence-associated β-galactosidase staining cells. D: Expression of genes of EPCs and BMSCs. $^{*}p<0.05$, $^{**}p<0.01$

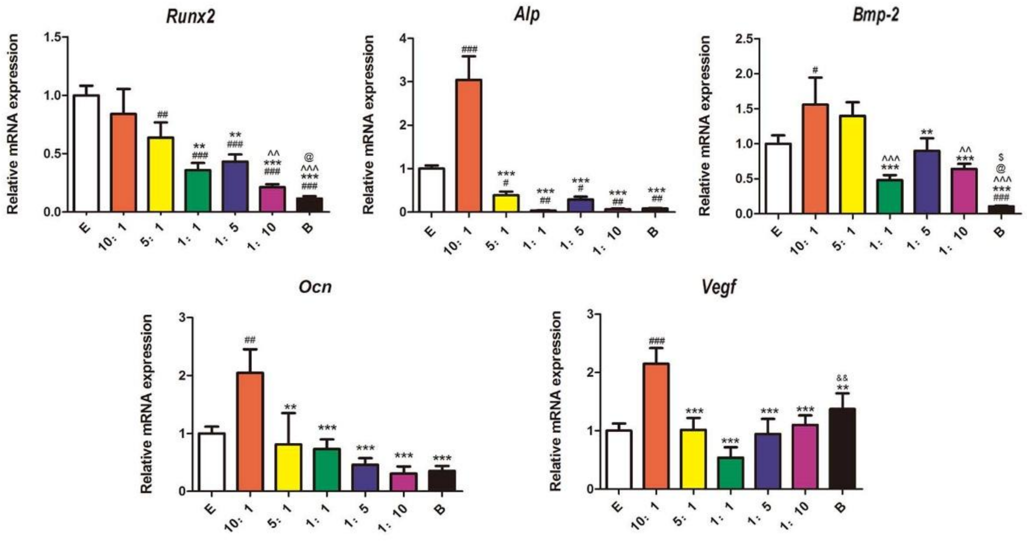

Supplementary Fig. S2. Expression of genes of co-cultured EPCs and BMSCs of different ratios

$^{\#}$p<0.05, $^{\#\#}$p<0.01, $^{\#\#\#}$p<0.001*vs* EPC, **p<0.01,***p<0.001 *vs* 10:1, $^{\wedge}$p<0.05, $^{\wedge\wedge}$ p<0.01, $^{\wedge\wedge\wedge}$ p<0.001*vs* 5:1, $^{\&\&}$p<0.01 *vs* 1:1, $^{@}$ p<0.05 *vs* 1:5, $^{\$}$ p<0.05 *vs* 1:10

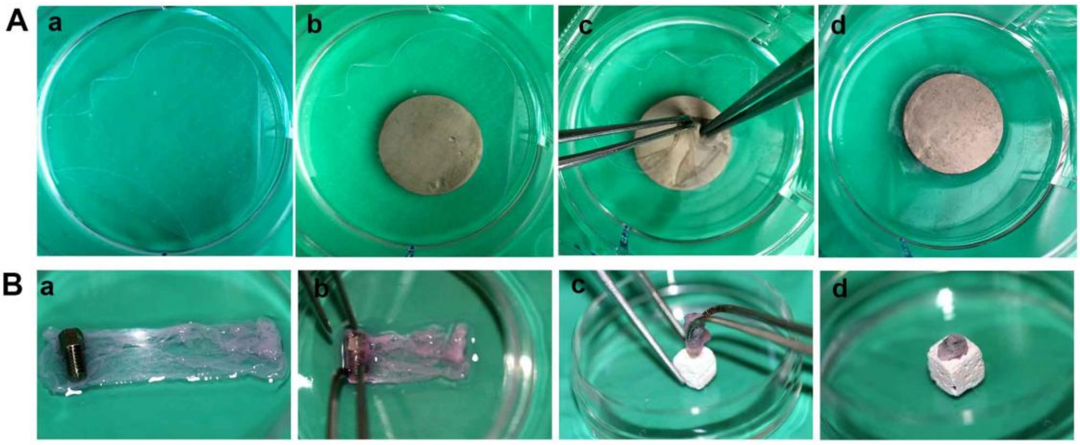

Supplementary Fig. S3. A: Fabrication of cell sheet-Ti disc complex. a: Detach cell sheet from plates. b, c: Wrap Ti disc with cell sheet. d: Turn over the complex. **B: Fabrication of cell sheet-implant complex.** a: Fold the cell sheet according to the length of the implant. b: Wrap the implant with cell sheet. c, d: Put the complex into the β-TCP cube.

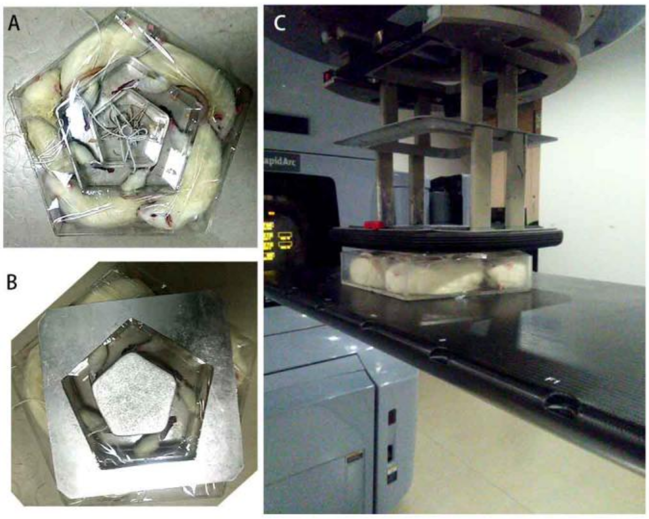

Supplementary Fig. S4. Radiation of rats. A: Position of rats for irradiation. B: Protection of other parts of rats. C: Radiotherapy setup.

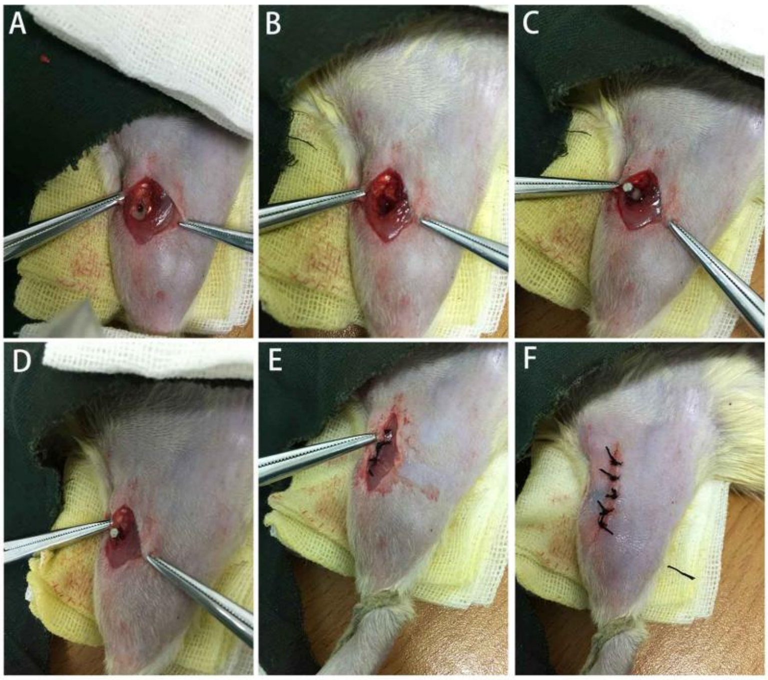

Supplementary Fig. S5. Procedure of implant surgery. A：The prepared implant sites. B: A layer of cell sheet was put inside the implant site. C, D: An implant wrapped with a piece of cell sheet of the same group was screwed in. E, F: Muscle tissue and skin were sutured separately.
